# Statistical Parsimony Networks and Species Assemblages in Cephalotrichid Nemerteans (Nemertea)

**DOI:** 10.1371/journal.pone.0012885

**Published:** 2010-09-21

**Authors:** Haixia Chen, Malin Strand, Jon L. Norenburg, Shichun Sun, Hiroshi Kajihara, Alexey V. Chernyshev, Svetlana A. Maslakova, Per Sundberg

**Affiliations:** 1 Institute of Evolution and Marine Biodiversity, Ocean University of China, Qingdao, China; 2 Department of Zoology, University of Gothenburg, Gothenburg, Sweden; 3 Sven Lovén Centre for Marine Sciences, Tjärnö, Sweden; 4 Department of Invertebrate Zoology, National Museum of Natural History, Smithsonian Institution, Washington, D.C., United States of America; 5 Department of Natural History Sciences, Faculty of Science, Hokkaido University, Sapporo, Japan; 6 Far East Division, A.V. Zhirmunsky Institute of Marine Biology, Russian Academy of Sciences, Vladivostok, Russia; 7 Oregon Institute of Marine Biology, University of Oregon, Charleston, Oregon, United States of America; American Museum of Natural History, United States of America

## Abstract

**Background:**

It has been suggested that statistical parsimony network analysis could be used to get an indication of species represented in a set of nucleotide data, and the approach has been used to discuss species boundaries in some taxa.

**Methodology/Principal Findings:**

Based on 635 base pairs of the mitochondrial protein-coding gene cytochrome *c* oxidase I (COI), we analyzed 152 nemertean specimens using statistical parsimony network analysis with the connection probability set to 95%. The analysis revealed 15 distinct networks together with seven singletons. Statistical parsimony yielded three networks supporting the species status of *Cephalothrix rufifrons*, *C. major* and *C. spiralis* as they currently have been delineated by morphological characters and geographical location. Many other networks contained haplotypes from nearby geographical locations. Cladistic structure by maximum likelihood analysis overall supported the network analysis, but indicated a false positive result where subnetworks should have been connected into one network/species. This probably is caused by undersampling of the intraspecific haplotype diversity.

**Conclusions/Significance:**

Statistical parsimony network analysis provides a rapid and useful tool for detecting possible undescribed/cryptic species among cephalotrichid nemerteans based on COI gene. It should be combined with phylogenetic analysis to get indications of false positive results, i.e., subnetworks that would have been connected with more extensive haplotype sampling.

## Introduction

Species delimitation is emerging as a major topic in current systematics [1], [2]. Although it is generally accepted that species constitute lineages [1], [3], it is a taxonomic challenge to recognize and delimit the species with traditional character sets and analytical procedures [1], [4], [5]. Although traditional methods used to delimit species are often morphology-based, progress in molecular techniques has led to increasing use of DNA data (e.g., [2], [6]–[8]). DNA taxonomy [9] and DNA barcoding (e.g., [10], [11]) have accelerated the rate at which new species are discovered and described [2].

Hart and Sunday [12] found empirically that subnetworks in statistical parsimony analysis [13] as implemented in the TCS program [14] coincided significantly with Linnaean names. The program calculates the maximum number of mutational steps constituting a parsimonious connection between two haplotypes with the probability of 95%, and then joins haplotypes into networks following algorithms in Templeton *et al.* [13]. Haplotypes separated by more mutational steps (i.e., the probability of secondary mutations exceeds 5%) remain disconnected. Hart and Sunday's [12] results suggest that statistical parsimony analysis could be used to differentiate species, and thus to detect species in a nucleotide-sequence dataset.

Nemerteans are traditionally identified and classified on morphological criteria, but the relatively low number of qualitative characters in general, and the paucity of species-specific characters, make species delimitation problematic, especially when comparing closely related species (e.g., [15]). Therefore, it would be useful to have in addition a DNA-based system for species identification, and for discovering assemblages of nemerteans comprising species. Here we follow the suggestion by Hart and Sunday [12] and test whether statistical parsimony network analysis based on mitochondrial cytochrome *c* oxidase I (COI) sequence can be used to recognize species within the palaeonemertean family Cephalotrichidae. This is a good target for such a study because species identification and delimitation of cephalotrichids is hampered by subtle morphological differences that often are difficult to describe unambiguously [16]–[18], which has led to a confusing taxonomic literature, thereby further complicating the issue of species delimitation in this taxon. Externally, most cephalotrichids look alike: body pale or translucent, whitish, yellowish, flesh-or straw-colored, with some species exhibiting a distinct coloration of the cephalic tip (e.g., [19]). Other key characters used to delimit species in the group are subjectively evaluated and their taxonomic/systematic significance is controversial. Contraction artifacts during fixation further impede species identification and delimitation using internal anatomy in these soft-bodied animals (e.g., [20], [21]). Therefore, it would not be surprising for cephalotrichids to contain numerous cryptic or unrecognized species, and, for specimens belonging to the same species to be assigned different names (especially if they originated from different geographical areas). The family currently contains 30 named species worldwide in one genus [22], [23] and we suspect several cases of undescribed and unnamed species. The sampled specimens were allocated to different putative species when possible, based on external characters, and others put down as “sp”. All specimens were codified from living material – ordinary fixation procedures would basically remove all external species characteristics.

## Results

The 152 aligned COI sequences were 635 bp long, of which 295 positions were variable and 274 parsimony informative. The mean base frequencies were as follows: A = 0.230, C = 0.148, G = 0.205 and T = 0.417.

**Figure 1:**
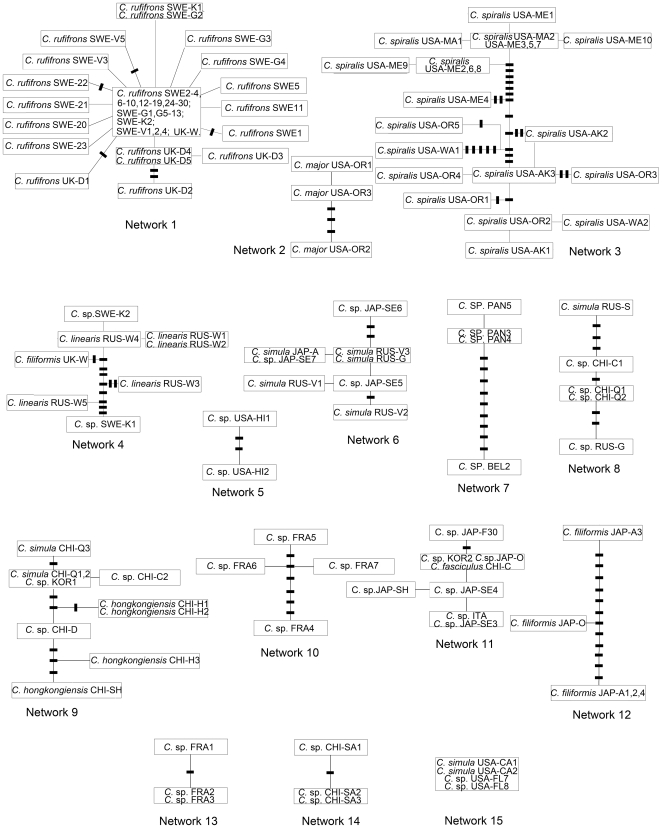
Haplotype networks for the COI gene in this study under the 95% parsimony criterion. Each bar indicates one missing unsampled haplotype. Two or more names in one frame indicate identical genotypes. The names in frames correspond to those labcodes in Table S1 (Species name plus the abbreviation of the locality and number). Abbreviations as per Table S1.

The 152 specimens comprised 90 unique haplotypes, and the statistical parsimony analysis revealed 15 distinct networks (Figure 1) and seven haplotypes that could not be connected to any of the other haplotypes (see Table S1, Networks 16–22). Three of the 15 networks corresponded entirely and exclusively with Linnaean names: *Cephalothrix rufifrons*, *C. major* and *C. spiralis*. Of these, the largest Network 1 (*C. rufifrons*) included 56 specimens from Sweden and England with 17 haplotypes. Network 2 (*C. major*) contained three haplotypes found in three individuals from southern Oregon coast, USA. Network 3 (*C. spiralis*) contained 17 haplotypes from 22 individuals collected from both the Pacific (Alaska, Washington, Oregon) and the Atlantic coast (Maine and Massachusetts) of USA. Genetic variation within these three networks/species is highest among the *C. major* specimens (Network 2), and lowest among the *C. rufifrons* (Network 1) specimens (Table 1).

**Table 1:** Number of individuals, haplotypes and haplotype diversity (*h*) in each haplotype network (Networks 1–15) and mean nucleotide Kimura-2-parameters (K2P) distances for COI gene within haplotype network.

Haplotype network	No. individuals	Haplotypes	Haplotype diversity (*h*)	K2P distance	
				Range	mean
Network 1	56	17	0.542	0.0000–0.0111	0.00151
Network 2	3	3	1.000	0.0016–0.0079	0.00528
Network 3	22	17	0.961	0.0000–0.0274	0.01500
Network 4	8	7	0.964	0.0000–0.0160	0.00939
Network 5	2	2	1.000	–	0.00474
Network 6	8	6	0.929	0.0000–0.0095	0.00390
Network 7	4	3	0.833	0.0000–0.0159	0.00849
Network 8	5	4	0.900	0.0000–0.0095	0.00554
Network 9	10	7	0.911	0.0000–0.0144	0.00667
Network 10	4	4	1.000	0.0032–0.0111	0.00713
Network 11	8	5	0.857	0.0000–0.0063	0.00276
Network 12	5	3	0.700	0.0000–0.0192	0.01103
Network 13	3	2	0.667	0.0000–0.0032	0.00211
Network 14	3	2	0.667	0.0000–0.0032	0.00211
Network 15	4	1	0.000	0.0000–0.0000	0.00000
Overall Average					0.00571

There is no strict correspondence between taxonomic names and remaining networks, although a clear geographical pattern emerges. Correspondingly, field notes often indicate uncertainty about identification of the specimens. A morphotype sometimes was designated based on a general or specific resemblance to a described species – e.g., those with yellow or orange snout designated as “*simula*” – with the expectation that these could comprise multiple species. Network 4 connects *C. filiformis* from Wales, UK with several haplotypes of *C. linearis* from the White Sea, Russia and the other two undescribed/unidentified cephalotrichid (*C.* sp. SWE-K1 and *C.* sp. SWE-K2) from Koster, Sweden. The remaining three haplotypes of *C. filiformis* (all from Japanese waters) constituted Network 12. Eleven specimens collected by different persons from six localities in four countries: USA, Russia, Japan and China were recorded as morphotype – not species – *C. simula* (*C. simula* USA-CA1–2, RUS-S, RUS-G, RUS-V1–3, JAP-A, CHI-Q1–3 in Table S1). Field notes indicated some doubts about identification, and statistical parsimony analysis separated specimens assigned this name into four networks (Networks 6, 8, 9, 15). Network 9 contained several specimens of *C. simula* and *C.* sp. from China and Korea, as well as all of the *C. hongkongiensis* from Hong Kong and Shenzhen, China. All others assigned to the *C. simula* morphotype group with unidentified species/specimens. Two such individuals from California, USA (*C. simula* USA-CA1, 2) shared an identical haplotype with two unknown cephalotrichids (*C.* sp. USA-FL7 and *C.* sp. USA-FL8) from Fort Pierce, Florida, USA (network 15). Network 6 contained *C. simula* haplotypes from the Russian side of the Sea of Japan, as well as Japanese specimens from Akkeshi and Seto. Specimens with haplotypes in network 8 roughly fit the description of *C. simula* (Table S1) and originate from the Russian side of the Sea of Japan, Sakhalin Island, Russia and, Qingdao and Changdao, China. Network 11 contained five haplotypes from seven unidentified specimens and one individual recorded as *C. fasciculus,* which shared an identical haplotype with two specimens from Jeju Island, Korea and Oshoro, Japan, respectively. One of the included specimens (*C.* sp. JAP-F30) in this network is from the type locality for *C. simula*. All of the specimens in Network 11 are from the Pacific Ocean (China, Korea and Japan), except for one, which was collected in Trieste, Italy.

The remaining multiple haplotype networks (5, 7, 10, 13, and 14) contained unassigned cephalotrichids. All of these networks are geographically cohesive. Network 5 contained two haplotypes from Hawaii. The Caribbean Network 7 included three closely related haplotypes from Bocas del Toro, Panama and a divergent haplotype from Belize. Network 10 contained four haplotypes from Roscoff, France. Network 13 contained two haplotypes from Roscoff, France. Finally, Network 14 consisted of specimens from Sanya, China. The next 6 networks (16–21) were from unidentified specimens collected in Spain (*C*. sp. SPA), Panama (*C*. sp. PAN 1, 2), Seto, Japan (*C*. sp. JAP-SE1, 2), and Vietnam (*C*. sp.VIE). Haplotype 22 contained a single specimen identified as *C. fasciculus* from Fukue, Japan.

The genetic distances (K2P) within and between the networks are listed in Table 1 and Table 2. The mean distances within networks are less than 1.5% (minimum 0.126%), while inter-network distances are above 4.3%, with a maximum of 32.0% (between Network 4 and Network 7).

**Table 2:** Kimura-2-parameters (K2P) distances for COI gene between haplotype networks/species.

	N1	N2	N3	N4	N5	N6	N7	N8	N9	N10	N11	N12	N13	N14	N15	N16	N17	N18	N19	N20	N21	N22
N1	-	21.1	18.3	20.8	15.2	15.7	28.1	16.6	16.1	7.20	16.5	19.9	16.1	24.1	17.2	11.0	16.4	16.1	17.2	28.0	15.4	15.1
N2		-	22.4	21.5	19.7	19.6	24.7	20.5	18.8	21.1	20.0	22.6	19.3	20.8	21.9	18.6	17.8	18.4	20.2	22.9	22.1	20.0
N3			-	10.6	16.5	15.3	31.4	15.0	16.8	17.3	15.3	12.1	15.9	25.9	16.9	16.5	17.9	15.6	16.1	30.1	16.1	17.5
N4				-	17.8	16.7	32.0	16.4	17.9	21.6	16.8	12.4	17.6	24.5	17.8	20.3	18.7	17.1	18.1	30.3	18.8	18.6
N5					-	16.2	29.7	16.3	17.0	17.3	17.5	21.0	16.1	25.6	17.5	17.4	17.5	17.7	18.5	27.4	18.3	18.6
N6						-	27.4	5.20	9.20	15.5	4.60	16.2	14.3	21.2	12.1	15.1	16.0	13.8	14.9	24.7	12.4	11.8
N7							-	27.6	27.4	28.8	24.9	29.2	27.4	31.0	27.3	28.0	26.2	27.5	28.5	13.4	26.6	25.6
N8								-	8.30	16.6	5.80	16.7	14.2	23.6	12.8	15.6	15.5	13.0	15.8	24.5	13.5	11.6
N9									-	16.4	8.60	18.5	13.4	23.9	12.2	16.0	16.2	14.2	16.7	25.2	13.0	13.4
N10										-	16.1	19.1	15.4	25.6	17.1	11.0	16.2	16.0	16.4	28.5	14.9	15.5
N11											-	15.8	14.2	23.7	12.1	16.3	15.0	13.1	16.3	25.2	11.6	11.3
N12												-	18.4	26.4	18.3	17.1	18.8	18.2	18.7	30.1	17.7	19.8
N13													-	25.2	16.2	15.9	16.9	16.4	14.7	27.4	13.6	16.8
N14														-	23.2	23.8	23.4	23.6	26.1	28.9	25.0	24.0
N15															-	15.6	19.2	10.9	15.7	27.9	13.8	11.6
N16																-	16.2	16.2	15.4	27.3	15.6	15.6
N17																	-	17.2	14.7	27.3	17.4	17.0
N18																		-	14.6	27.0	15.0	12.5
N19																			-	27.3	15.7	16.7
N20																				-	28.3	27.3
N21																					-	13.4
N22																						-

N1–22 indicates Networks 1–22 (Networks 1–14, See Figure 1); N15, *C*. *simula* CA1 (*C. simula* CA2, *C*. sp. FL7, *C*. sp. FL8); N16, *C*. sp. SPA; N17–18, *C*. sp. PAN1, 2; N19–20, *C*. sp. JAP-SE1, 2; N21, *C*. sp. VIE; N22, *C*. *fasciculus* JAP-F33. Abbreviations as per Table S1.

**Figure 2:**
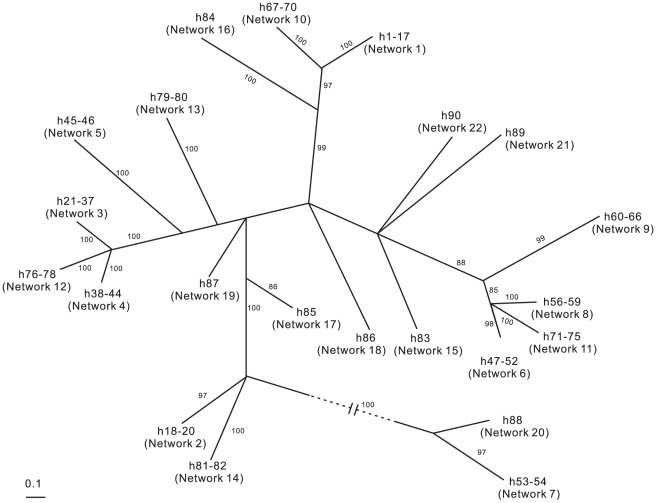
Unrooted cladogram from maximum likelihood analysis of the 90 unique haplotypes of 152 COI sequences. Branch support values (>80%) are shown on the branches. Abbreviations as per Table S1. Branch lengths are proportional to genetic distance, branch lengths to Networks 7 (h53–54) and 20 (h88) are drawn to 1/3 of actual length for convenience.

The phylogeny estimated based on maximum likelihood revealed 22 supported clades corresponding to the subnetworks in the statistical parsimony analysis (unrooted haplotype tree in Figure 2).

## Discussion

*Cephalothrix rufifrons*, *C. spiralis* and *C. major* were coherently placed in one network each. These species are characteristic in external appearance, having been well described (or re-described). The real genetic diversity appears to correspond to what has been figured out by human visual interpretation based on external appearances. Specimens identified as *C. rufifrons* and *C. major* in the present analysis are only from limited geographical area, whereas those identified as *C. spiralis* are from both Atlantic and Pacific coasts of USA. The other named and unnamed specimens were separated into different networks, each containing specimens similar in appearance collected from the same geographical region (with a few notable exceptions). This suggests that statistical parsimony networks of COI sequences can be used successfully to assign nemertean specimens to species, as has been done previously for other taxa [7], [25]. The networks could be used to corroborate known species and aid in the discovery of new ones, as well as guide more detailed morphological/ecological studies to evaluate species status.

*Cephalothrix linearis* originally was described as *Planaria linearis* by Rathke [26] from the North Sea coast of Denmark, and *C. filiformis* originally was described as *Planaria filiformis* by Johnston [27] from the British Isles. The two species are similar in external appearances, but *C. filiformis* was described as contracting into a tight spiral when disturbed. Internally, it possesses an inner circular muscle layer in the foregut region [19] that is reported as absent in *C. linearis*. The statistical parsimony haplotype analysis indicates that *C. filiformis* from Wales UK and *C. linearis* from White Sea, Russia (in Network 4) constitute one species, supporting Bürger's [28] suggestion to synonymize them. This conclusion is further supported by the low intra-network variation (0.5% to 1.3%). This begs the question of whether an inner circular muscle is an autapomorphy for *C. filiformis*, or whether we, in fact, have not sampled any specimens from the species once named *C. linearis*.

The five specimens from Japan, identified as *C. filiformis*, formed a distinct network (12). Sequence divergence based on the pairwise K2P distances between the Japanese specimens (Network 12) and the Northern European (Network 4) is 12.4% (Table 2). In *C. filiformis* [27] the mid-dorsal blood vessel runs along the proboscis, instead of the rhynchocoel wall. In *C. filiformis* from Japan, described by Iwata [29], the vessel is not attached to the rhynchocoel wall, but is pendent in the rhynchocoel lumen [30]. The occurrence in Japanese waters is quite outside the range of this species based on other records from the British Isles, the coast of France, and northern Spain [19], [22].

In the phylogram (Figure 2), these two networks (4 and 12) form a well supported polytomy with haplotypes from Network 3, which includes exclusively specimens identified as *spiralis* (from east and west coast of North America). This pattern would indicate that these haplotypes could belong to the same species, with a wide geographic distribution. Coe [31] when describing *C. spiralis* admits that it “is evidently closely related to *C. filiformis* found on the coasts of Great Britain and northern France”. The taxonomy of these species is confused and it is difficult to decide whether the phylogram is correct in connecting these names into one species, leading to a false positive in the network analysis caused by undersampled haplotype diversity. Considering the wide geographic range sampled when it comes to these three networks, we are inclined to consider the subnetworks to represent different species even though the genetic distances between these subnetworks are in the lower end (10.6–12.4%) of the entire ranges (Table 2). The assigned names do not then correspond to original descriptions.

*Procephalothrix simulus* was described from Fukue, Japan by Iwata [32] and reported to lack a horizontal muscle plate between the rhynchocoel and the alimentary tract, but Iwata reported the presence of this feature in a different specimen from Akkeshi, Japan, in later work [29]. Our study includes 11 specimens from six geographically distinct sampling sites, identified as, or characterized as similar to *C. simula* based on external morphology. These specimens are similar to each other in being pale or translucent whitish, yellowish, or greenish color with several exhibiting a distinctly orange or yellowish cephalic tip. Some differ only in the color of the body and cephalic patch. Statistical parsimony analysis separates these specimens into four networks, and the K2P distance ranges from 0% to 12.7% between individual specimens. The short inter-specimen distances (0–1.436%) between *C. hongkongiensis* from Hong Kong and Shenzhen and those specimens (accordingly) misidentified as *C. simula* from Qingdao (CHI-QD1–3), China clearly suggest all members in Network 9 belong to the same species.

The sister clade to Network 9 in the phylogram (Figure 2) contains haplotypes from the three networks (6, 8 and 11). Most of these haplotypes/specimens are unidentified. The two networks (6 and 8) contain “*simula*” individuals, one from China (Network 8) and the other from Japan (Network 6), with overlapping distribution in the Russian coast of the Sea of Japan. The inter-network distances between 6, 8 and 11 are in comparison low (Table 2: 4.6% between Network 6 and 11, 5.2% between Network 6 and 8, and 5.8% between Network 8 and 11). We conclude, based on the combination of a supported clade in the phylogram (Figure 2), low inter-network distances, and restricted geographic distribution, that these subnetworks contain haplotypes from the same species. The separation into three subnetworks in the statistical parsimony analysis would then be the result of undersampling the intraspecific haplotype variation, leading to a false positive result.

Specimens in Network 7 are morphologically close to *Cephalothrix alba* (Gibson and Sundberg, 1992), in bearing a row of epidermal, lateral, cephalic ocelli, making the morphotype relatively easy to distinguish from other cephalotrichids in this study. This could indicate that the specimens in Network 7 should be labeled *C. alba*, especially since this morphotype appears to have a global distribution (JLN, pers. obs.). But, we have currently COI data only for the Caribbean region, while *C. alba* was originally described from Hong Kong.

The family Cephalotrichidae currently contains 30 described species [22], [23]. Whether some or any of the unnamed specimens placed into distinct haplotype networks by TCS program correspond to previously described species remains to be tested. They were intentionally not attributed to known species because morphology indicated something deviant from known species — suggesting that they are undescribed — or the collector considered the available diagnoses for some described species to be inadequate.

Cephalotrichid nemerteans are exclusively marine benthic dwellers. With respect to the distribution of the specimens in the present study, most of them are from limited regions, such as Europe (*C. rufifrons*, Network 4), North America (*C. spiralis*, *C. major*), and Northwest Pacific (Networks 6, 7, 8, 9, 12). However, the sample sizes are unequal across localities and small for some of them, and it is premature to make conclusions about the species ranges and patterns of distribution.

The statistical parsimony approach provides a useful tool for detecting putative undescribed species and situations where we need to examine the taxonomy of nemerteans more closely. Most of the cephalotrichid nemerteans are inherently difficult to identify using characters of external appearance. In this respect, the analytical approach we have applied has many advantages, for example it provides a rapid partitioning into Linnaean species and facilitates the identification of cryptic and undescribed species. Recent studies (e.g., [21], [33], [34]) show accumulating evidence of cryptic species among nemerteans, and the shortcomings of morphology when it comes to the systematics and taxonomy of this group. This study shows that DNA-based methods and approaches have a useful role in analyzing taxonomic problems, and that statistical parsimony analysis is a fast and efficient method to indicate possible species assemblages in need of further examination. The interpretation of our analyses is affected by modest and uneven sampling and we cannot with confidence distinguish between inadequate lineage sorting and true species delimitations. This is not for lack of collecting effort; it reflects the inherent difficulty in collecting nemerteans, as well as the limited number of people and opportunities to do so. Thus, the results in this study trigger the beginning of further studies of the included specimens, and extended taxon sampling needed to fill recognized geographic gaps. Even if the TCS method strongly suggests new species, these need to be tested by added geographical, ecological, and phylogenetic analyses. The results do confirm that in the aim of estimating the true species diversity in a poorly known phylum, the network approach is an efficient tool that will show, for example, what groups or species suffer from major taxonomy flaws or misinterpretations.

## Materials and Methods

### Specimens and DNA extraction

A total of 152 specimens sequenced are listed in Table S1, together with collectors, GenBank accession numbers and the collection areas/sites. The data set contains 43 specimens that could not be confidently placed in any of the described species based on morphology or geographical location alone. Collectors' comments on these specimens are included.

Specimens were preserved in 70–95% ethanol, or a solution of dimethyl sulphoxide, disodium EDTA, and saturated NaCl [35], after examination and identification based on external characters. DNA was extracted using the QIAamp DNA Mini Kit (Qiagen) or a standard phenol-chloroform protocol [36].

### Amplification and sequencing

The partial COI mtDNA sequence was amplified using universal primers LCO1490 and HCO2198 [37]. Polymerase chain reactions (PCRs) were carried out in a 25-µl reaction mixture with final concentrations of 10 mM Tris-HCl, 50 mM KCl, 2 mM MgCl_2_, 0.3 mM of each primer, 100 mM of each dNTP, 1 unit of *rTaq* polymerase (TaKaRa) and 2 µl DNA template, or the standard recommendations of Illustra™ PuReTaq Ready-To-Go PCR Beads kit from GE. Thermal cycling was performed in MyCycler™ (BioRad), programmed for an initial denaturing step of 94°C for 5 min, followed by 35 cycles at 94°C for 40 s, 48°C for 45 s, 72°C for 60 s, and final extension at 72°C for 8 min.

PCR products were evaluated with agarose gel electrophoresis and purified with the E.Z.N.A. Cycle-Pure Kit (Omega Biotek), or with the PCR Gel extraction kit (Takara). Sequencing was performed by the genetic service facilities of Macrogen (Korea) or Invitrogen (Shanghai, China) with the same primer pairs as initial PCRs.

### Sequence analysis

All sequence data were checked against the original chromatograms using Bioedit v. 7.0.1 [38] and aligned with Clustal X 1.83 [39] with default parameters. The nucleotide composition and Kimura's 2-parameter (K2P) genetic distance [40] between networks were calculated in MEGA 4.0 [41].

### COI haplotype network

A statistical parsimony analysis [13] was conducted with all individual COI sequences from 152 specimens using the program TCS v.1.21 [14] to generate haplotype networks between closely related sequences, regardless of species category. This program also calculated the significant number of substitutions connecting haplotypes in the network according to the algorithm described in Templeton *et al.* [13]. The connection limit excluding homoplasic changes was set to 95% in accordance with Hart and Sunday [12].

### Cladistic analysis

We used the maximum likelihood (ML) criterion for studying the relationships between the haplotypes. ML analysis to estimate the haplotype phylogeny was performed with the program PhyML 3.0 [42]. Bootstrap values were determined from 1000 replicates. MODELTEST v.3.06 [43] was used to choose the substitution model for our data based on the Akaike information content. The selected model was GTR+I+G with proportion of invariable sites  = 0.438, and gamma shape parameter  = 0.501.

## Supporting Information

Table S1 List of cephalotrichids included in the analysis, localities, labcodes, collectors, haplotypes, comments on undescribe specimens and GenBank accession number.
(0.39 MB DOC)
