# Supplementary material for: Statistical Parsimony Networks and Species Assemblages in Cephalotrichid Nemerteans (Nemertea)
Source: PLoS One. 2010 Sep 21;5(9):e12885. doi: 10.1371/journal.pone.0012885 (PMC2943479; doi:10.1371/journal.pone.0012885)
Supplement: Table S1 — List of cephalotrichids included in the analysis, localities, labcodes, collectors, haplotypes, comments on undescribe specimens and GenBank accession number. (0.39 MB DOC) [file pone.0012885.s001.doc]

**Table S1.** List of cephalotrichids included in the analysis, localities, labcodes, collectors, haplotypes, comments on undescribe specimens and GenBank accession number.

| **Species** | **Locality** | **Labcode^a^** | **Collector^b^** | **Haplotype** | **Comments** | **Accession No.** |
| --- | --- | --- | --- | --- | --- | --- |
| Network 1 |  |  |  |  |  |  |
| *Cephalothrix rufifrons* (Johnston, 1837) | Bonden, Sweden | *C. rufifrons* SWE1 | PS | h1 |  | GU726713 |
| *Cephalothrix rufifrons* | Bonden, Sweden | *C. rufifrons* SWE2 | PS | h2 |  | GU726726 |
| *Cephalothrix rufifrons* | Bonden, Sweden | *C. rufifrons* SWE3 | PS | h2 |  | GU726727 |
| *Cephalothrix rufifrons* | Bonden, Sweden | *C. rufifrons* SWE4 | PS | h2 |  | GU726714 |
| *Cephalothrix rufifrons* | Humlesäcken, Sweden | *C. rufifrons* SWE5 | PS | h3 |  | GU726605 |
| *Cephalothrix rufifrons* | Skeppsholmen, Sweden | *C. rufifrons* SWE6 | PS | h2 |  | GU726606 |
| *Cephalothrix rufifrons* | Skeppsholmen, Sweden | *C. rufifrons* SWE7 | PS | h2 |  | GU726728 |
| *Cephalothrix rufifrons* | Skeppsholmen, Sweden | *C. rufifrons* SWE8 | PS | h2 |  | GU726715 |
| *Cephalothrix rufifrons* | Skeppsholmen, Sweden | *C. rufifrons* SWE9 | PS | h2 |  | GU726716 |
| *Cephalothrix rufifrons* | Skeppsholmen, Sweden | *C. rufifrons* SWE10 | PS | h2 |  | GU726717 |
| *Cephalothrix rufifrons* | Skeppsholmen, Sweden | *C. rufifrons* SWE11 | PS | h4 |  | GU726590 |
| *Cephalothrix rufifrons* | Skeppsholmen, Sweden | *C. rufifrons* SWE12 | PS | h2 |  | GU726718 |
| *Cephalothrix rufifrons* | Skeppsholmen, Sweden | *C. rufifrons* SWE13 | PS | h2 |  | GU726719 |
| *Cephalothrix rufifrons* | Skeppsholmen, Sweden | *C. rufifrons* SWE14 | PS | h2 |  | GU726720 |
| *Cephalothrix rufifrons* | Skeppsholmen, Sweden | *C. rufifrons* SWE15 | PS | h2 |  | GU726721 |
| *Cephalothrix rufifrons* | Skeppsholmen, Sweden | *C. rufifrons* SWE16 | PS | h2 |  | GU726722 |
| *Cephalothrix rufifrons* | Skeppsholmen, Sweden | *C. rufifrons* SWE17 | PS | h2 |  | GU726723 |
| *Cephalothrix rufifrons* | Stångholmen, Sweden | *C. rufifrons* SWE18 | PS | h2 |  | GU726724 |
| *Cephalothrix rufifrons* | Stångholmen, Sweden | *C. rufifrons* SWE19 | PS | h2 |  | GU726725 |
| *Cephalothrix rufifrons* | Stångholmen, Sweden | *C. rufifrons* SWE20 | PS | h5 |  | GU726591 |
| *Cephalothrix rufifrons* | Stångholmen, Sweden | *C. rufifrons* SWE21 | PS | h6 |  | GU726592 |
| *Cephalothrix rufifrons* | Stångholmen, Sweden | *C. rufifrons* SWE22 | PS | h7 |  | GU726593 |
| *Cephalothrix rufifrons* | Stångholmen, Sweden | *C. rufifrons* SWE23 | PS | h8 |  | GU726594 |
| *Cephalothrix rufifrons* | Stångholmen, Sweden | *C. rufifrons* SWE24 | PS | h2 |  | GU726729 |
| *Cephalothrix rufifrons* | Stångholmen, Sweden | *C. rufifrons* SWE25 | PS | h2 |  | GU726731 |
| *Cephalothrix rufifrons* | Stångholmen, Sweden | *C. rufifrons* SWE26 | PS | h2 |  | GU726732 |
| *Cephalothrix rufifrons* | Stångholmen, Sweden | *C. rufifrons* SWE27 | PS | h2 |  | GU726733 |
| *Cephalothrix rufifrons* | Stångholmen, Sweden | *C. rufifrons* SWE28 | PS | h2 |  | GU726734 |
| *Cephalothrix rufifrons* | Stångholmen, Sweden | *C. rufifrons* SWE29 | PS | h2 |  | GU726736 |
| *Cephalothrix rufifrons* | Stångholmen, Sweden | *C. rufifrons* SWE30 | PS | h2 |  | GU726737 |
| *Cephalothrix rufifrons* | Wembury/Salcombe, Devon, UK | *C. rufifrons* UK-D1 | JN | h9 |  | GU726601 |
| *Cephalothrix rufifrons* | Wembury/Salcombe, Devon, UK | *C. rufifrons* UK-D2 | JN | h10 |  | GU726602 |
| *Cephalothrix rufifrons* | Wembury/Salcombe, Devon, UK | *C. rufifrons* UK-D3 | JN | h11 |  | GU726603 |
| *Cephalothrix rufifrons* | Wembury/Salcombe, Devon, UK | *C. rufifrons* UK-D4 | JN | h12 |  | GU726604 |
| *Cephalothrix rufifrons* | Wembury/Salcombe, Devon, UK | *C. rufifrons* UK-D5 | JN | h12 |  | GU733828 |
| *Cephalothrix rufifrons* | Koster, Sweden | *C. rufifrons* SWE-K1 | MS | h13 |  | GU726595 |
| *Cephalothrix rufifrons* | Koster, Sweden | *C. rufifrons* SWE-K2 | MS | h2 |  | GU726738 |
| *Cephalothrix rufifrons* | Grötholmen, Sweden | *C. rufifrons* SWE-G1 | MS | h2 |  | GU733829 |
| *Cephalothrix rufifrons* | Grötholmen, Sweden | *C. rufifrons* SWE-G2 | MS | h13 |  | GU726688 |
| *Cephalothrix rufifrons* | Grötholmen, Sweden | *C. rufifrons* SWE-G3 | MS | h14 |  | GU726596 |
| *Cephalothrix rufifrons* | Grötholmen, Sweden | *C. rufifrons* SWE-G4 | MS | h15 |  | GU726597 |
| *Cephalothrix rufifrons* | Grötholmen, Sweden | *C. rufifrons* SWE-G5 | MS | h2 |  | GU726739 |
| *Cephalothrix rufifrons* | Grötholmen, Sweden | *C. rufifrons* SWE-G6 | MS | h2 |  | GU726740 |
| *Cephalothrix rufifrons* | Grötholmen, Sweden | *C. rufifrons* SWE-G7 | MS | h2 |  | GU726735 |
| *Cephalothrix rufifrons* | Grötholmen, Sweden | *C. rufifrons* SWE-G8 | MS | h2 |  | GU726741 |
| *Cephalothrix rufifrons* | Grötholmen, Sweden | *C. rufifrons* SWE-G9 | MS | h2 |  | GU726742 |
| *Cephalothrix rufifrons* | Grötholmen, Sweden | *C. rufifrons* SWE-G10 | MS | h2 |  | GU726743 |
| *Cephalothrix rufifrons* | Grötholmen, Sweden | *C. rufifrons* SWE-G11 | MS | h2 |  | GU726744 |
| *Cephalothrix rufifrons* | Grötholmen, Sweden | *C. rufifrons* SWE-G12 | MS | h2 |  | GU726745 |
| *Cephalothrix rufifrons* | Grötholmen, Sweden | *C. rufifrons* SWE-G13 | MS | h2 |  | GU726746 |
| *Cephalothrix rufifrons* | Vattenholmen, Sweden | *C. rufifrons* SWE-V1 | MS | h2 |  | GU726747 |
| *Cephalothrix rufifrons* | Vattenholmen, Sweden | *C. rufifrons* SWE-V2 | MS | h2 |  | GU726748 |
| *Cephalothrix rufifrons* | Vattenholmen, Sweden | *C. rufifrons* SWE-V3 | MS | h16 |  | GU726598 |
| *Cephalothrix rufifrons* | Vattenholmen, Sweden | *C. rufifrons* SWE-V4 | MS | h2 |  | GU726730 |
| *Cephalothrix rufifrons* | Vattenholmen, Sweden | *C. rufifrons* SWE-V5 | MS | h17 |  | GU726599 |
| *Cephalothrix* *rufifrons* | Wales, UK | *C. rufifrons* UK-W | MS | h2 |  | GU726632 |
| Network 2 |  |  |  |  |  |  |
| *Cephalothrix major* (Coe, 1930) | Oregon coast, USA | *C. major* USA-OR1 | MLS | h18 |  | GU726689 |
| *Cephalothrix major* | Oregon coast, USA | *C. major* USA-OR2 | MLS | h19 |  | GU726690 |
| *Cephalothrix major* | Oregon coast, USA | *C. major* USA-OR3 | MLS | h20 |  | GU726691 |
| Network 3 |  |  |  |  |  |  |
| *Cephalothrix spiralis*  (Coe, 1930) | Mt. Desert Isl., Maine, USA | *C. spiralis* USA-ME1 | JN | h21 |  | GU726697 |
| *Cephalothrix spiralis* | Mt. Desert Isl., Maine, USA | *C. spiralis* USA-ME2 | JN | h22 |  | GU726698 |
| *Cephalothrix spiralis* | Mt. Desert Isl., Maine, USA | *C. spiralis* USA-ME3 | JN | h23 |  | GU726699 |
| *Cephalothrix spiralis* | Mt. Desert Isl., Maine, USA | *C. spiralis* USA-ME4 | JN | h24 |  | GU726700 |
| *Cephalothrix spiralis* | Mt. Desert Isl., Maine, USA | *C. spiralis* USA-ME5 | JN | h23 |  | GU726701 |
| *Cephalothrix spiralis* | Mt. Desert Isl., Maine, USA | *C. spiralis* USA-ME6 | JN | h22 |  | GU726702 |
| *Cephalothrix spiralis* | Mt. Desert Isl., Maine, USA | *C. spiralis* USA-ME7 | JN | h23 |  | GU726703 |
| *Cephalothrix spiralis* | Mt. Desert Isl., Maine, USA | *C. spiralis* USA-ME8 | JN | h22 |  | GU726704 |
| *Cephalothrix spiralis* | Mt. Desert Isl., Maine, USA | *C. spiralis* USA-ME9 | JN | h25 |  | GU726705 |
| *Cephalothrix spiralis* | Mt. Desert Isl., Maine, USA | *C. spiralis* USA-ME10 | JN | h26 |  | GU726706 |
| *Cephalothrix spiralis* | Nahant, Massachusetts, USA | *C. spiralis* USA-MA1 | JN | h27 |  | GU726707 |
| *Cephalothrix spiralis* | Nahant, Massachusetts, USA | *C. spiralis* USA-MA2 | JN | h23 |  | GU726708 |
| *Cephalothrix spiralis* | Kachemak Bay, Alaska, USA | *C. spiralis* USA-AK1 | JN | h28 |  | GU726709 |
| *Cephalothrix spiralis* | Kachemak Bay, Alaska, USA | *C. spiralis* USA-AK2 | JN | h29 |  | GU726710 |
| *Cephalothrix spiralis* | Kachemak Bay, Alaska, USA | *C. spiralis* USA-AK3 | JN | h30 |  | GU726711 |
| *Cephalothrix spiralis* | San Juan Island, Washington, USA | *C. spiralis* USA-WA1 | JN | h31 |  | GU726712 |
| *Cephalothrix spiralis* | San Juan Island, Washington, USA | *C. spiralis* USA-WA2 | JN | h32 |  | GU726648 |
| *Cephalothrix spiralis* | Oregon coast, USA | *C. spiralis* USA-OR1 | SM | h33 |  | GU726692 |
| *Cephalothrix spiralis* | Oregon coast, USA | *C. spiralis* USA-OR2 | SM | h34 |  | GU726693 |
| *Cephalothrix spiralis* | Oregon coast, USA | *C. spiralis* USA-OR3 | SM | h35 |  | GU726694 |
| *Cephalothrix spiralis* | Oregon coast, USA | *C. spiralis* USA-OR4 | SM | h36 |  | GU726695 |
| *Cephalothrix spiralis* | Oregon coast, USA | *C. spiralis* USA-OR5 | SM | h37 |  | GU726696 |
| Network 4 |  |  |  |  |  |  |
| *Cephalothrix linearis*  (Rathke, 1799) | White Sea, Russia | *C. linearis* RUS-W1 | SM | h38 |  | GU726649 |
| *Cephalothrix linearis* | White Sea, Russia | *C. linearis* RUS-W2 | SM | h38 |  | GU726650 |
| *Cephalothrix linearis* | White Sea, Russia | *C. linearis* RUS-W3 | SM | h39 |  | GU726651 |
| *Cephalothrix linearis* | White Sea, Russia | *C. linearis* RUS-W4 | SM | h40 |  | GU726652 |
| *Cephalothrix linearis* | White Sea, Russia | *C. linearis* RUS-W5 | SM | h41 |  | GU726653 |
| *Cephalothrix* sp. | Koster, Sweden | *C*. sp. SWE-K1 | MS | h42 |  | GU726595 |
| *Cephalothrix* sp. | Koster, Sweden | *C*. sp. SWE-K2 | MS | h43 |  | GU726738 |
| *Cephalothrix filiformis*  (Johnston, 1828) | Wales, UK | *C. filiformis* UK-W | MS | h44 |  | EU489496^c^ |
| Network 5 |  |  |  |  |  |  |
| *Cephalothrix* sp. | Kaneohe, Hawaii, USA | *C*. sp. USA-HI1 | JN | h45 | Red head up to mouth, cluster of long cilia on tip of head – moustache; strange gonads, unilateral; 2 mm; coarse sand at 13 m depth. | GU726634 |
| *Cephalothrix* sp. | Kaneohe, Hawaii, USA | *C*. sp. USA-HI2 | JN | h46 | Same as above | GU726633 |
| Network 6 |  |  |  |  |  |  |
| *Cephalothrix simula* | Akkeshi Bay, Japan | *C. simula* JAP-A | JN | h47 |  | AJ436945^c^ |
| *Cephalothrix* sp. | Seto, Japan | *C*. sp. JAP-SE5 | JN | h48 | Pale brownish or greenish yellow, with orange or yellow on snout or over much of head; pale white annuli around body; contracts linearly; up 70 mm relaxed; from calcareous algae. | GU726663 |
| *Cephalothrix* sp. | Seto, Japan | *C*. sp. JAP-SE6 | JN | h49 | Same as above | GU726664 |
| *Cephalothrix* sp. | Seto, Japan | *C*. sp. JAP-SE7 | JN | h47 | Same as above | GU726665 |
| *Cephalothrix simula* | Vostok Bay, Sea of Japan, Russia | *C. simula* RUS-V1 | AC | h50 | Body length reaches up to 5–6 cm long and is characterized by having an orange tip of the head. | GU726641 |
| *Cephalothrix simula* | Vostok Bay, Sea of Japan, Russia | *C. simula* RUS-V2 | AC | h51 | Same as above | GU726642 |
| *Cephalothrix simula* | Vostok Bay, Sea of Japan, Russia | *C. simula* RUS-V3 | AC | h52 | Same as above | GU726643 |
| *Cephalothrix simula* | Peter the Great Bay, Russia | *C. simula* RUS-G | AC | h52 | Body length about 20 cm, pale yellowish-orange (or pale orange). Anterior tip pale reddish. Intestine not dark. | GU726609 |
| Network 7 |  |  |  |  |  |  |
| *Cephalothrix* sp. | Bocas del Toro, Panama | *C*. sp. PAN3 | JN | h53 | Described as *C*. sp. BEL2. Up to 20mm long, subtidal in fine to medium coarse sand, 3-30 m depth. | GU726677 |
| *Cephalothrix* sp. | Bocas del Toro, Panama | *C*. sp. PAN4 | JN | h53 | Same as above | GU726678 |
| *Cephalothrix* sp. | Bocas del Toro, Panama | *C*. sp. PAN5 | JN | h54 | Same as above | GU726679 |
| *Cephalothrix* sp. | Carrie Bow Cay, Belize | *C*. sp. BEL2 | JN | h55 | Grayish white with irregular row of ~25 black integumentary ocelli on each side of head reaching mouth, anterior ocelli form a cluster; lateral epidermal bristles along Same as above region; body-wall and rhynchocoelic septa; fragments easily, contracts linearly, proboscis papillae packed with cluster of parallel elongate rhabdite-like granules (60–100/papilla); subterminal proboscis pore, 6 mm long fragment; from medium-fine sand on reef ridge at 15m | GU726682 |
| Network 8 |  |  |  |  |  |  |
| *Cephalothrix simula* | Sakhalin island, Russia | *C. simula* RUS-S | AC | h56 | Body length about 30 cm; anterior body portion pale yellowish-orange, anterior tip intensive orange; intestine with olive tint; testes whitish. | GU726607 |
| *Cephalothrix* sp. | Qingdao, Shandong, China | *C.* sp. CHI-Q1 | HC | h57 | Body dull green, anterior tip yellowish orange. | GU726624 |
| *Cephalothrix* sp. | Qingdao, Shandong, China | *C.* sp. CHI-Q2 | HC | h57 | Same as above | GU726625 |
| *Cephalothrix* sp. | Changdao, Shandong, China | *C.* sp. CHI-C1 | HC | h58 | Body olive, white laterally (color of mature gonads); laboratory observation unavailable (specimens died during transportation). | GU726618 |
| *Cephalothrix* sp. | Peter the Great Bay, Russian | *C.* sp. RUS-G | AC | h59 | Body length about 6 cm, whitish with pale olive tint, anterior tip pale orange-yellow. | GU726608 |
| Network 9 |  |  |  |  |  |  |
| *Cephalothrix hongkongiensis*  Sundberg, Gibson and Olsson, 2003 | Starfish Bay, Hong Kong, China | *C. hongkongiensis* CHI-H1 | SS | h60 | Body pale yellow, somewhat translucent, yellowish orange cephalic patch visible but very pale. | GU726611 |
| *Cephalothrix hongkongiensis* | Starfish Bay, Hong Kong, China | *C. hongkongiensis* CHI-H2 | SS | h60 | Same as above | GU726612 |
| *Cephalothrix hongkongiensis* | Starfish Bay, Hong Kong, China | *C. hongkongiensis* CHI-H3 | SS | h61 | Same as above | GU726613 |
| *Cephalothrix simula* Iwata, 1952 | Qingdao, Shandong, China | *C. simula* CHI-Q1 | HC | h62 | Similar to Hong Kong specimens; orange cephalic patch apparent in most specimens, but not so distinct in the others. Abundant in some habitats. | GU726626 |
| *Cephalothrix simula* | Qingdao, Shandong, China | *C. simula* CHI-Q2 | HC | h62 | Same as above | GU726627 |
| *Cephalothrix simula* | Qingdao, Shandong, China | *C. simula* CHI-Q3 | HC | h63 | Same as above | GU726628 |
| *Cephalothrix* sp. | Changdao, Shandong, China | *C*. sp. CHI-C2 | SS | h64 | Anterior 1/3 pale yellowish orange, paler posteriorly; tip of head orange. | GU726614 |
| *Cephalothrix hongkongiensis* | Shenzhen, Guangdong, China | *C*. *hongkongiensis* CHI-SH | SS | h65 | Similar to Hong Kong specimens, but cephalic patch indistinct in most specimens. | GU726610 |
| *Cephalothrix* sp. | Dachen Island, Zhejiang, China | *C*. sp. CHI-D | SS | h66 | Similar to the *C. simula* CHI-Q1-3.Without a distinct orange cephalic patch. | GU726617 |
| *Cephalothrix* sp. | Jeju Island, Korea | *C*. sp. KOR1 | AC | h62 | Body length 5-6 cm, whitish yellow, “head” pale yellow. | GU726644 |
| Network 10 |  |  |  |  |  |  |
| *Cephalothrix* sp. | Roscoff, France | *C*. sp. FRA4 | SM | h67 | Pinkish-yellow to orange body. A bright spot (sometimes two lateral spots can be distinguished) of orange pigment near the tip of the head. | GU726673 |
| *Cephalothrix* sp. | Roscoff, France | *C*. sp. FRA5 | SM | h68 | Same as above | GU726674 |
| *Cephalothrix* sp. | Roscoff, France | *C*. sp. FRA6 | SM | h69 | Same as above | GU726675 |
| *Cephalothrix* sp. | Roscoff, France | *C*. sp. FRA7 | SM | h70 | Same as above | GU726676 |
| Network 11 |  |  |  |  | Same as above |  |
| *Cephalothrix* sp. | Fukue, Japan | *C.* sp. JAP-F30 | HK | h71 | Cephalic tip yellow; foregut dark olive green; intestine orange. | GU726622 |
| *Cephalothrix* sp. | Jeju Island, Korea | *C*. sp. KOR2 | AC | h72 | Body length 3–5 cm, yellowish (intestine more dark), anterior tip reddish. mouth protruded and not far behind the brain. | GU726646 |
| *Cephalothrix* sp. | Oshoro, Japan | *C.* sp. JAP-O | HK | h72 | Cephalic tip translucent; pre-oral region long, reddish dark salmon in color; basement body translucent; alimentary canal light salmon. | GU726619 |
| *Cephalothrix* sp. | Shimoda, Japan | *C.* sp. JAP-SH | HK | h73 | Cephalic tip orange red; pre-oral region and foregut region khaki-goldenrod; intestine dark orange. | GU726620 |
| *Cephalothrix* sp. | Seto, Japan | *C.* sp. JAP-SE3 | JN | h74 | Very pale translucent greenish-yellow body, with apparent body “septation”, shortens linearly but tail region tends not to contract, head cylindrical, body slightly flattened, 20 mm, shallow subtidal from brown seaweed. | GU726661 |
| *Cephalothrix* sp. | Seto, Japan | *C.* sp. JAP-SE4 | JN | h75 | Same as above | GU726662 |
| *Cephalothrix* sp. | Trieste, Italy | *C.* sp. ITA | SM | h74 | Whole individual and magnified anterior end semitranslucent pre-oral region and opaque greenish post-oral region. | GU733830 |
| *Cephalothrix fasciculus*  Iwata, 1952 | Changdao, Shandong, China | *C. fasciculus* CHI-C | SS | h72 |  | GU726615 |
| Network 12 |  |  |  |  |  |  |
| *Cephalothrix filiformis* | Oshoro, Japan | *C.* *filiformis* JAP-O | HK | h76 |  | GU726637 |
| *Cephalothrix filiformis* | Akkeshi Bay, Japan | *C*. *filiformis* JAP-A1 | JN | h77 |  | GU726635 |
| *Cephalothrix filiformis* | Akkeshi Bay, Japan | *C*. *filiformis* JAP-A2 | JN | h77 |  | GU726636 |
| *Cephalothrix filiformis* | Akkeshi Bay, Japan | *C*. *filiformis* JAP-A3 | JN | h78 |  | GU726645 |
| *Cephalothrix filiformis* | Akkeshi Bay, Japan | *C*. *filiformis* JAP-A4 | JN | h77 |  | AJ436944^c^ |
| Network 13 |  |  |  |  |  |  |
| *Cephalothrix* sp. | Roscoff, France | *C*. sp. FRA1 | SM | h79 | Characterized by pinkish-yellow body. As opposed to the species *C*. sp. FRA4-7, there is no orange pigment concentrated on the tip of the head | GU726670 |
| *Cephalothrix* sp. | Roscoff, France | *C*. sp. FRA2 | SM | h80 | Same as above | GU726671 |
| *Cephalothrix* sp. | Roscoff, France | *C*. sp. FRA3 | SM | h80 | Same as above | GU726672 |
| Network 14 |  |  |  |  |  |  |
| *Cephalothrix* sp. | Sanya, Hainan, China | *C*. sp. CHI-SA1 | SS | h81 | Body white or with a yellowish tinge; somewhat translucent; head slightly pointed without any color patterns; mouth far away from the anterior tip (about 10% of body length). Most likely an undescribed species. | GU726629 |
| *Cephalothrix* sp. | Sanya, Hainan, China | *C*. sp. CHI-SA2 | SS | h82 | Same as above | GU726630 |
| *Cephalothrix* sp. | Sanya, Hainan, China | *C*. sp. CHI-SA3 | SS | h82 | Same as above | GU726631 |
| Network 15 |  |  |  |  |  |  |
| *Cephalothrix simula* | San Diego, California, USA | *C. simula* USA-CA1 | JN | h83 | Orange on snout and extending over head; creamy to pale yellowish brown body; up to 60mm relaxed; in crustose red algae, other compact red algae, and kelp holdfasts on tidal and barely subtidal rocky ledge; contracts linearly. | GU726639 |
| *Cephalothrix simula* | San Diego, California, USA | *C. simula* USA-CA2 | JN | h83 | Same as above | GU726640 |
| *Cephalothrix* sp. | Fort Pierce, Florida, USA | C. sp. USA-FL7 | JN | h83 | Orange snout; from subtidal sand in sparse seagrass bed and dock ropes with fouling community in full-salinity estuarine inlet; up to 80 mm relaxed. | GU726668 |
| *Cephalothrix* sp. | Fort Pierce, Florida, USA | C. sp. USA-FL8 | JN | h83 | Same as above | GU726669 |
| Network 16 |  |  |  |  |  |  |
| *Cephalothrix* sp. | Armintza, Bizkaia, Spain | *C*. sp. SPA | SM | h84 | Whitish. | GU726616 |
| Network 17 |  |  |  |  |  |  |
| *Cephalothrix* sp. | Bocas del Toro, Panama | *C*. sp. PAN1 | JN | h85 | True interstitial; very adhesive, translucent almost colorless, ~1m depth in medium-coarse sand; fragmenting and deteriorating, few observations. | GU726680 |
| Network 18 |  |  |  |  |  |  |
| *Cephalothrix* sp. | Bocas del Toro, Panama | *C*. sp. PAN2 | JN | H86 | White, “typical” cephalotrichid, medium sand, 10mm, not active. | GU726681 |
| Network 19 |  |  |  |  |  |  |
| *Cephalothrix* sp. | Seto, Japan | *C.* sp. JAP-SE1 | JN | h87 | Spirals when irritated, resembles slightly flattened *C. spiralis*; brain tinged red, gut whitish, 20 mm; subtidal (5m?) sediment off Shisozima, Seto. | GU726667 |
| Network 20 |  |  |  |  |  |  |
| *Cephalothrix* sp. | Seto, Japan | *C.* sp. JAP-SE2 | JN | h88 | Subtidal under rock off Seto, fragmenting single specimen, no time for observations. | GU726666 |
| Network 21 |  |  |  |  |  |  |
| *Cephalothrix* sp. | Vietnam | *C.* sp. VIE | AC | H89 | Body length about 7 cm, orange-yellow, with two longitudinal dark brown dotted strips and 38 thin rings with brown pigment; anterior tip orange; eyes absent. | GU726621 |
| Network 22 |  |  |  |  |  |  |
| *Cephalothrix* *fasciculus* | Fukue, Japan | *C. fasciculus* JAP-F33 | HK | h90 |  | GU726623 |

^a^ Species name plus the abbreviation of the locality and number. *Abbreviations:*

AK=Alaska, USA; BEL=Belize; CA=California, USA; CHI=China; FRA= France; HI=Hawaii, USA; ITA=Italy; JAP=Japan; KOR=Korea; MA=Massachusetts, USA; ME=Maine, USA; SWE=Sweden; OR=Oregon coast, USA; PAN=Panama; RUS=Russia; VIE=Vietnam; WA=Washington, USA;

^b^ AC=Alexey Chernyshev, JN=Jon Norenburg, HC=Haixia Chen, HK=Hiroshi Kajihara, MLS=Megan Schwartz, MS=Malin Strand, PS=Per Sundberg, SM=Svetlana Maslakova, SS=Shichun Sun;

^C^ [24].
